# Order, Disorder, and Everything in Between

**DOI:** 10.3390/molecules21081090

**Published:** 2016-08-19

**Authors:** Shelly DeForte, Vladimir N. Uversky

**Affiliations:** 1 Department of Molecular Medicine, Morsani College of Medicine, University of South Florida, Tampa, FL 33612, USA; sdeforte@health.usf.edu; 2 USF Health Byrd Alzheimer’s Research Institute, Morsani College of Medicine, University of South Florida, Tampa, FL 33612, USA; 3 Laboratory of Structural Dynamics, Stability and Folding of Proteins, Institute of Cytology, Russian Academy of Sciences, St. Petersburg 194064, Russia

**Keywords:** unfolded, flexible, unstructured, intrinsically disordered, protein function, structural heterogeneity, multi-functionality

## Abstract

In addition to the “traditional” proteins characterized by the unique crystal-like structures needed for unique functions, it is increasingly recognized that many proteins or protein regions (collectively known as intrinsically disordered proteins (IDPs) and intrinsically disordered protein regions (IDPRs)), being biologically active, do not have a specific 3D-structure in their unbound states under physiological conditions. There are also subtler categories of disorder, such as conditional (or dormant) disorder and partial disorder. Both the ability of a protein/region to fold into a well-ordered functional unit or to stay intrinsically disordered but functional are encoded in the amino acid sequence. Structurally, IDPs/IDPRs are characterized by high spatiotemporal heterogeneity and exist as dynamic structural ensembles. It is important to remember, however, that although structure and disorder are often treated as binary states, they actually sit on a structural continuum.

## 1. The Dominant Paradigms in Protein Science

The dominant paradigms in protein science were, in many ways, shaped by the earliest experiments in the field. Those early experiments were constrained by many of the same limitations we have today. Can this protein be purified? Does it have consistency and simplicity in its function? Ultimately, the measures of success in the dominant methods of experimentation can direct scientific thought regarding what is most important in the study of proteins. As an example, early protein studies in the nineteenth century revolved around the easily obtained and easily crystallized protein hemoglobin. Myosin, also easily available and identified around the same time as hemoglobin, was largely ignored because it was not crystalline. Because of this, it would be another 100 years before we understood Myosin at even the most basic level [1].

Jacob Berzelius coined the term “catalysis” in 1836 [2], amidst intense interest in enzymes, which at that point had not yet been shown to be proteins. Emil Fischer demonstrated enzyme specificity, and established his seminal lock and key model in 1894 [3], a model that still dominates our understanding of enzyme catalysis today. Interestingly, Fischer also proposed that proteins would prove to have a maximum length of 4000 amino acids [1], demonstrating the intuitive, and incorrect, speculations that follow from a strict adherence to the lock and key model. Experimental confirmation of the structure-function relationship continued with the Anfinsen’s demonstration that RNase A could be re-natured in vitro from the completely unfolded state with an accompanying restoration of function [4], followed by early X-ray crystal studies of enzymes such as lysozyme [5] and ribonuclease-S [6]. It is undeniable that the success of these early experiments helped to shape dominant ideas of well-behaved protein behavior, where well-behaved was synonymous with well-structured, with one singular function, and one mechanism of action.

In many ways, these early experiments represented canonical examples of how proteins should be, which all further experiments were then held against. Therefore, protein behaviors that ran counter to the expected results were considered anomalies. That a protein could take an extended form with minimal residual structure was well understood due to numerous denaturation experiments. However, it was assumed that the native and functional state of a protein must have a stable structure. Therefore, results that ran counter to this assumption were typically considered a problem with the experiment or the experimenter, and not a result of the intrinsic properties of the protein. As these anomalies accumulated to the point where they could no longer be ignored, these problem proteins and problem regions were often seen as functionally irrelevant, and in many cases, removed before experimentation.

## 2. Defining Intrinsically Disordered Proteins

**Figure 1:**
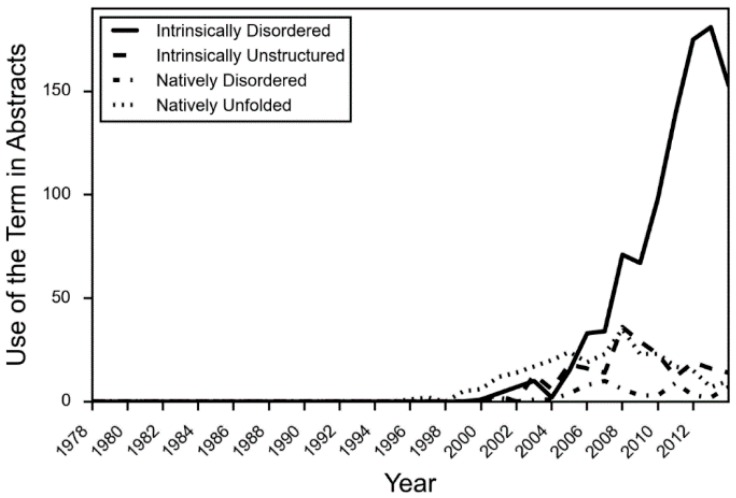
The usage of IDP terminology in PubMed abstracts. The occurrence of each IDP term was counted for each year in abstracts of articles in PubMed associated with 1127 known IDPs.

Even while the structure-function paradigm was strengthened in protein science, examples of intrinsically disordered proteins (IDPs) regularly appeared in the literature. Prompted by the increased application of optical rotary dispersion in the 1950s and 1960s to the investigation of protein structure, Jirgensons suggested a classification scheme that included a category called “disordered” [7]. By this time, phosphvitin [7], casein [8], and histones [9] had been shown to have unusual structural properties. In 1971, it was proposed that two regions of missing electron density in the X-ray crystal structure of staphylococcus nuclease were “disordered” [10] as well. However, as more IDPs were uncovered, a wider variety of terms were applied to describe the phenomenon. Tau was initially referred to as “natively denatured” [11], while α-synuclein was called “natively unfolded” [12]. Early reviews and theoretical work in the field used various terms as well, such as “intrinsically unstructured” [13], “natively disordered” [14] and “loopy” [15], among others. Vague terms such as “flexible” [16] and “mobile” [17] have a long history of hiding in the literature as well. In fact, until about 2005, the four most common terms “intrinsically disordered”, “intrinsically unstructured”, “natively disordered”, and “natively unfolded”, were all used about equally in the literature (Figure 1) [18]. However, after that time, due in part to a concerted effort in the field to use consistent terminology, the term “intrinsically disordered” became the predominant and agreed upon term.

The field of un-structural biology has arisen to try to explain all cases of proteins that fall outside of the structure–function paradigm, and it is necessarily broad in scope because of this. Therefore, just as there have been challenges in reaching a consensus on terminology, there have also been similar challenges in defining protein intrinsic disorder. However, despite these challenges, several common definitions have emerged.

Different definitions of IDPs emphasize different experimental and theoretical perspectives. An IDP may be described as having little or no ordered secondary or tertiary structure. This definition emphasizes an IDP’s difference from proteins as understood by the tools of structural biology. An IDP may also be described as under-folded, or as failing to fold independently. This definition emphasizes that an IDP may have the same physical properties as the unfolded state or as a folding intermediate of an ordered protein, such as random coil, molten globule, or pre-molten globule. Finally, it has become increasingly common to focus on the ensemble nature of IDPs when defining them. This places IDPs in the context of behavior that can increasingly be measured by NMR. The properties of IDPs are typically described as being present in vivo, in vitro, or under functional conditions. This is to emphasize that IDPs display their structural properties in a functional, native state.

It is interesting to note that all of these definitions do not place disordered proteins in a single opposite position from ordered proteins, but instead place ordered and disordered proteins at different points on a continuum. This is clearer, when we understand that both ordered and disordered proteins have movement at the atomic level and about their Ramachandran angles. However, in ordered proteins this motion is sufficiently small that a consensus position can be inferred. On the other hand, an intrinsically disordered protein has movement that precludes the collapse into a single point, both at the ensemble and individual protein level.

From these definitions, one can extrapolate a theoretical definition based on theories of protein folding. If a protein folds into its lowest energy conformation, then we can define an IDP as a protein that does not have a single global minimum in conformational space [19] or, alternately, IDPs can be described as having a relatively flat free energy surface [20]. Unfortunately, this theoretical definition cannot currently be characterized experimentally for structured or disordered proteins. 

Finally, it is necessary to distinguish proteins that are mostly or fully disordered from proteins with isolated regions of disorder. The term IDP is used to refer to proteins that are fully disordered, or contain long, defining regions of disorder. In contrast, when a protein is mostly structured but displays some regions of disorder, it is said to have intrinsically disordered protein regions (IDPRs). Proteins that contain a mix of ordered and disordered regions are also called hybrid proteins.

## 3. The Subtler Side of Disorder

In much the same way that protein science has been shaped by the dominant experimental methods, so too has the field which specializes in studying protein intrinsic disorder. Early measurements of protein intrinsic disorder were obtained by low-resolution methods such as optical rotary dispersion and circular dichroism. These methods cannot measure individual regions of disorder, but only the structural properties over the whole protein. X-ray crystallography can indicate small regions of possible disorder by their absence in the resolved three-dimensional structure, but cannot establish the cause. Therefore, the early emphasis in the field was on proteins that are mostly or fully disordered in vitro or in vivo, such as Myelin Basic Protein [21], α-synuclein [12], MAP2 [22], and tau [11].

**Figure 2:**
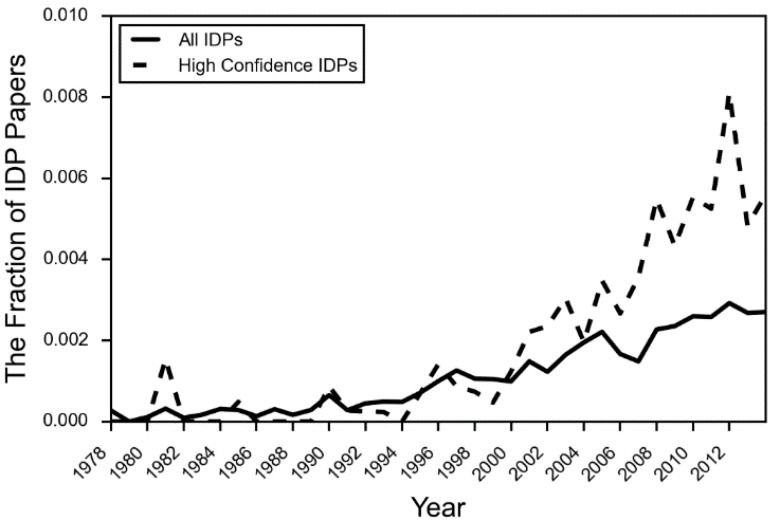
The fraction of PubMed IDs using IDP terminology by year. The fraction for each year is calculated by the number of PubMed IDs associated with IDPs that use IDP language, divided by the total number of PMIDs associated with the IDP proteins in the set. High confidence IDPs are those that have an extensive amount of experimental evidence verifying that the protein is intrinsically disordered.

Aside from the experimental challenges, there are additional reasons why the IDP literature has remained focused on highly and consistently disordered proteins. Acceptance of the relevance, and even the existence of IDPs and IDPRs has not yet solidified in the literature. Discussions of IDPs in textbooks are still largely absent, with a few exceptions in the last five years [23]. The citation aggregator PubMed did not add “intrinsically disordered proteins” to its MeSH (Medical Subject Headings) terms until 2014. The number of papers in the body of literature covering IDPs that actually use IDP terminology is still a fraction of a percent (Figure 2). Despite this, the rise of NMR and sequence-based bioinformatics methods has greatly expanded the experimental and theoretical literature on the topic of IDPs [23]. We now have the experimental tools to begin to characterize subtler categories of disorder, such as conditional disorder and partial disorder.

**Figure 3:**
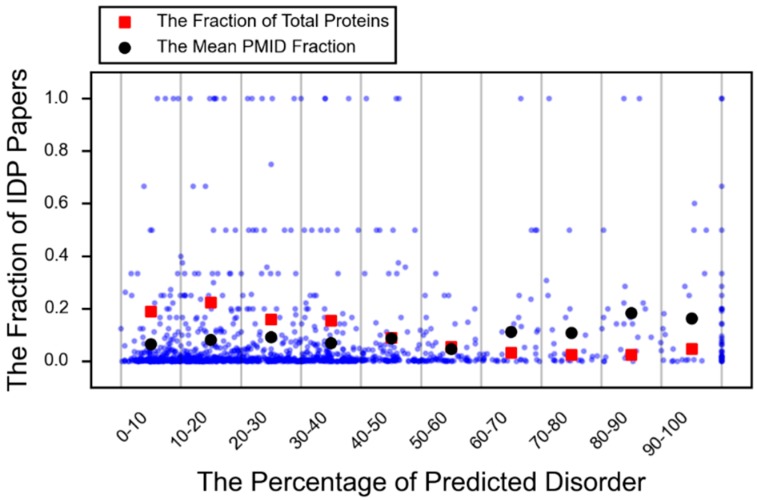
The fraction of predicted disorder versus the fraction of PubMed IDs that use IDP language. Each blue dot represents a protein. The percent predicted disorder is plotted against the fraction of PubMed IDs that use intrinsic disorder language divided by all PubMed IDs associated with that protein search term. For each fraction of predicted disorder interval (0%–10%, 10%–20%, etc.), the fraction of the total proteins in that interval is plotted in red. The mean of the fraction of disorder PubMed IDs is plotted for each fraction of disorder interval in black.

A protein that is conditionally disordered is either ordered or disordered based on the environmental context or interaction partner. It is a term that encompasses both disorder-to-order transitions and transient (or cryptic) disorder, which is functionally relevant disorder that arises from structured regions (order-to-disorder) [24]. Furthermore, an increasing number of examples of small, but important IDPRs are appearing in the literature as can be seen in Figure 3 (red square).

## 4. The Thin Line between Order and Disorder

Of special interest are proteins that display the properties of both structure and disorder, specifically proteins that have been at least partially crystallized, and enzymes, which will have a structured catalytic region in most cases. While structure and disorder are often treated as binary states, they actually sit on a continuum. Since many proteins might exist in the middle of the structure–disorder continuum, it is useful to identify the conceptual line between a structured and disordered protein or protein region, and explore why it is necessary to use the tools and language of disorder when structure is present. 

When attempting to semantically separate structure from disorder, it becomes clear that neither the term “structured” nor “disordered” is precisely correct. All proteins have some movement, and no protein is completely chaotic. Because these are conceptual frameworks that do not point to precise biological realities, the tools of structural or un-structural biology should be applied when most useful to solve the problem. The separating line between order and disorder is therefore drawn not by theoretical descriptions, but by practical considerations.

When a protein can no longer be adequately described by a single three-dimensional structure or a series of snapshots in three dimensions, then the language of disorder has now become useful. IDPs are defined by conformational uncertainty, and are typically characterized by a combination of sequence level features and a description of the overall shape, i.e., extended, random coil, molten globule, or pre-molten globule. Additionally, an IDP that changes shape must also be described using the axis of time, and it is along this axis that structural biology and un-structural biology most acutely diverge. The introduction of a time variable greatly increases the possibilities when describing IDPs and IDPRs, and it is change over time that allows us to describe the mechanisms that IDPs may employ and the advantages imparted by disorder.

As an example, short segments of disorder are commonly observed in the form of hinges that move a domain in a controlled way, or loops that have an open and closed conformation, such as the WPD loop in the bacterial protein tyrosine phosphatase YopH [25]. While these regions are technically disordered, the ability to describe the movement as a series of structural snapshots typically places these dynamic movements within the realm of structural biology. However, other small disordered segments called Molecular Recognition Features (MoRFs) [26], which undergo a contextual transition between disorder and order upon binding, have a function that is defined by the presence of disorder and the transition to an ordered state, and not by a specifically defined three-dimensional structure. Therefore, even despite their short lengths and disorder–order transitions, MoRFs fall within the realm of un-structural biology.

Furthermore, whether a protein is considered to be an IDP or to have an IDPR is largely determined by the functional significance assigned to the disorder. Disordered regions that have no known function are often considered to be functionally neutral sequence noise. An a priori assumption that a disordered region is function-neutral can create circular support for itself if this region is removed before experimentation; therefore, the identification of disorder specific functions is of critical importance. 

## 5. The Mechanisms of Disorder

A great deal of experimental and theoretical work has been done in order to illuminate how IDPs and IDPRs fit within a functional protein universe. When viewed within the context of finely regulated interaction and signaling networks, where proteins may need to display multiple context dependent behaviors, the advantages of disorder begin to become clear. While many historical examples of the functional properties of IDPs and IDPRs have come from studies of non-enzymes, an increasing number of more recent studies have shown that these functional advantages are also demonstrable in enzymes.

### 5.1. Entropy

**Figure 4:**
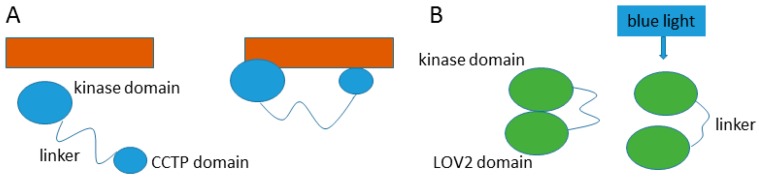
Entropic chain functions. (**A**) Yck2 (in blue) uses a disordered interdomain linker to bind to two separate domains on Akr1 (in orange); (**B**) The disordered interdomain linker in Phototropin 2 (in green) becomes elongated when irradiated with blue light, causing the activating LOV2 domain to separate from the kinase domain.

Entropic functions make use of the advantages inherent in dynamic movement within a disordered protein chain. Entropic chains can provide precise spacing between functional domains, creating a less restricted search space, maintaining separation between domains, or creating the opportunity for two or more domains to interact with each other, or with another partner. Two enzymes with kinase domains demonstrate the utility of a disordered interdomain linker. The kinase Yck2 has a long disordered interdomain linker that allows the kinase domain and a conserved *C*-terminal peptide (CCTP) domain to interact with two separate Akr1 domains simultaneously [27], while a disordered interdomain linker in Phototropin 2 becomes elongated when exposed to blue light irradiation, preventing the LOV2 domain from making contact with and activating the kinase domain [28] (Figure 4). Additionally, there are non-enzyme examples of entropic clock functions, such as the voltage-gated potassium channel of nerve axons which uses a ball and chain mechanism to inactivate the channel [29]. Entropic bristles use entropy to fill space as is seen in the gating of the nuclear pore complex through the repulsion caused by disordered nucleoporins [30].

### 5.2. Accessibility

Posttranslational modification (PTM) requires site accessibility, so it is therefore not surprising that many PTM sites are embedded in disordered regions, which can provide a large surface area with a limited number of residues. Phosphorylation sites in particular have been shown to be enriched in disordered residues [31]. Most well-known IDPs have phosphorylation sites, and they have been demonstrated in the IDPRs of enzymes as well. For example, the intrinsically disordered Cap region of both Abl and Arg non-receptor tyrosine kinases is rich in phosphorylation sites that regulate multiple domains [32].

Site accessibility is also required for proteolytic processing that generates protein fragments with altered activity. For example, the phosphatase calcineurin contains an intrinsically disordered regulatory domain that is susceptible to proteolytic cleavage in vitro [33] and in vivo [34] that significantly increases its activity.

### 5.3. Plasticity

Some of the most striking functional advantages of IDPs and IDPRs come from their ability to contextually change due to binding or environmental cues. IDPs and IDPRs may change shape in response to interactions with proteins, nucleic acids, and other ligands, allowing them to specifically bind to a wide variety of partners. For example, the disordered loop near the active site of Mitochondrial 2,4-Dienoyl-CoA reductase allows this enzyme to accommodate a wide range of fatty acids [35]. Protein Kinase R has two intrinsically disordered interdomain regions that may allow it to effectively dimerize when interacting with a RNA activators of varying size and shape [36]. Plasticity may also be helpful in identifying and negotiating disordered regions in substrates, and appears to play an important role in ubiquitination pathways. For instance, E3 ubiquitin ligases that bind both substrates and E2 ubiquitin conjugating enzymes are significantly more disordered than those that engage in single interactions [37].

## 6. Disorder-Related Biological Functions

The biophysical mechanisms used by disordered regions are disproportionately connected to particular biological roles, such as signaling and molecular and cellular regulation [38]. This is not surprising, considering the ability of disordered regions to change over time and to adapt based on the environmental context.

### 6.1. Signaling 

Signaling pathways provide a way for complicated biological systems to coordinate physiological activities and responses. Signaling pathways can usually be described as a linear cascade of interactions that triggers some kind of change in the cell. Frequently PTMs such as phosphorylation play a key role in this, and disorder frequently plays a role both in the accessibility of PTM sites, and the activity of kinases [39]. Key sequence signals may be encoded in disordered sequences, such as in sulfhydryl oxidase ALR which has an IDPR that acts as a mitochondrial targeting signal in the cytosol and a recognition site in the disulfide relay system of the intermembrane space [40]. Receptors, which are often a starting point for a signaling cascade, may have IDPRs that expose phosphorylation sites or bind to signaling molecules as is seen in the receptor tyrosine-protein kinase ErbB2 [41]. 

### 6.2. Regulation

IDPs are frequently involved in regulation at the cellular level through involvement in gene transcription [42] and protein degradation [37,43], and at the protein level, through allosteric effects or PTMs that result in the masking and unmasking of interaction sites. As an example, phosphorylation of the IDP 4E-BP2 acts as a regulatory switch by inducing a disorder-order transition and preventing binding with eIF4E [44]. Conversely, regulation of glucokinase is facilitated by an order-disorder transition that causes a time-delay when glucose is low [45]. 

IDPs are also abundant in protein degradation pathways. There are a number of E3 ubiquitin-protein ligases which have long stretches of disorder that appear to mediate interactions with a variety of mostly disordered substrates [37]. For example, San1 is an E3 ubiquitin-protein ligase which has extended disorder in its N and C terminal substrate binding regions. Interestingly, San1 avoids auto-ubiquitination through the absence of lysines in its disordered binding regions [46]. Ubiquitin-independent protein degradation pathways also involve disordered protein regions. The enzymes thymidylate synthase and ornithine decarboxylase both contain IDPRs that appear to contain the sole requirements for ubiquitin independent degradation [43].

## 7. Disorder and Protein Evolution

The evolution of protein coding regions in genomes presents a fundamental mystery. The human genome, the genome of the flowering plant *Arabidopsis thaliana,* and the genome of the protozoa *Tetrahyma* are all estimated to have approximately 27,000–29,000 protein coding regions. On the other hand, *Danio rerio* (zebrafish) and *Mus musculus* have close to 40,000 protein coding regions each [47]. It is clear that protein coding regions do not scale linearly with organismic complexity. One compelling explanation for this is that protein intrinsic disorder combined with alternative splicing facilitates tightly regulated and context specific multi-functional behavior that allows more complicated organisms to make the most of a limited genome [42,48].

There are several pieces of evidence to support a hypothesis of evolutionarily directed functional disorder. Bioinformatics analyses show that eukaryotes are more disordered than prokaryotes [49], natural sequences are more disordered than random sequences even with the same amino acid composition [50], and disorder within natural sequences is non-random in its patterns [51]. Disordered regions tend to evolve more rapidly while maintaining their physiological functions [52], therefore, functional disorder may provide an advantage by buffering a genome against mutations. Finally, it can be argued that complex signaling networks and finely tuned regulatory mechanisms are themselves a response to organismic complexity; therefore, the overrepresentation of IDPs and IDPRs in signaling and regulation also supports the hypothesis of the directed evolution of disorder in genomes. Interestingly, some protists have more disorder than multicellular eukaryotes, suggesting there may be an optimal amount of disorder for an organism that is partly based on lifestyle [49].

## 8. The Tools of the Un-Structural Biologist

**Figure 5:**
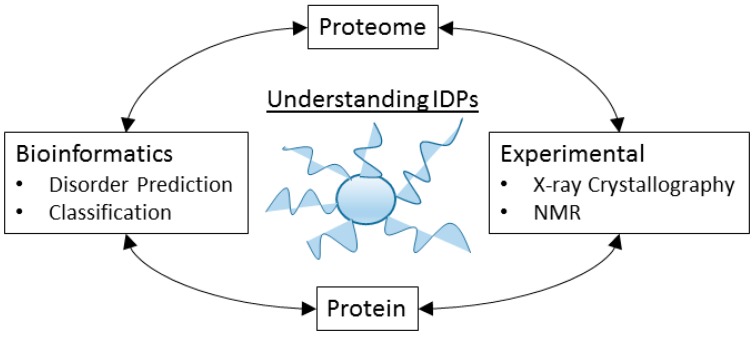
Experimental and bioinformatics techniques work together to describe the properties of disorder in proteomes and proteins.

The study of IDPs and IDPRs requires a large number of experimental and computational methods, and typically the results of these experiments combine together to form a picture of the disorder properties over the protein and the proteome (Figure 5).

### 8.1. Some Experimental Techniques

#### 8.1.1. X-ray Crystallography

It is somewhat surprising that X-ray crystal structures provide one of the largest datasets of experimentally indicated IDPRs, considering that X-ray crystallography is one of the primary tools of structural biology. However, missing regions in X-ray crystal structures are often caused by IDPRs. The challenge with using this data, however, is that missing regions are an imperfect indication of protein intrinsic disorder, as there are multiple possible explanations for a missing region, including experimental artifacts or annotation errors. Furthermore, authentic IDPRs identified in X-ray crystal structures will be non-representative in terms of the size of the region and the amino acid composition, due to their emergence from a very structured set of proteins. The decision to use X-ray crystal structure data as an indication of disorder must therefore be made by balancing the usefulness of a large amount of data against the imperfections in the data. 

#### 8.1.2. Nuclear Magnetic Resonance

Nuclear Magnetic Resonance (NMR) is arguably the current best experimental technique for identifying protein intrinsic disorder and conformational ensembles. The key differences that make NMR superior to X-ray crystallography for identifying IDPs is that NMR does not require crystallization and NMR can provide direct observation of disorder instead of simply indicating a lack of structure. However, there are limitations in the size of the protein that restrict the applicability of NMR, and the amount of NMR data is still significantly less than the amount of X-ray crystal structure data. 

Identifying IDPs and IDPRs using NMR can be accomplished through several different approaches. A collapsed HSQC NMR spectrum will indicate disorder over the entire protein, whereas a dispersed spectrum will indicate a structured protein. NMR techniques can also be used to generate conformational ensembles and multiple methods can be employed to measure the differences between ensembles [53,54]. Additionally, chemical shift and ^15^N (^1^H) NOE data can provide flexibility information without the requirement for any structural models [55]. 

#### 8.1.3. Combining Experimental Techniques

The number of experimental techniques that can be used to study IDPs is extensive, and in fact multiple books [56,57] and reviews [58,59] have been dedicated to this topic. In practice, multiple techniques of varying resolution are typically employed and the aggregated evidence is used to create models of the disordered regions. These include low-resolution spectroscopic techniques such as circular dichroism, optical rotary dispersion, Fourier-transform infrared spectroscopy, and deep-UV resonance Raman spectroscopy. Additionally, the level of protein compaction can be measured by small angle X-ray scattering, small angle neutron scattering, gel-filtration, and viscometry. The properties of individual protein molecules can help identify ensemble properties and can be obtained via high speed atomic force microscopy (AFM), and single-molecule fluorescence resonance energy transfer (SM-FRET).

### 8.2. Bioinformatics Analysis

Bioinformatics tools and analysis have played a large part in the study of IDPs and the establishment of the field. The tools used to study IDPs typically focus on extracting information from protein sequences, however, genome studies focusing on the evolution of IDPs and the enrichment of splicing sites in disordered regions are common as well [60,61]. Several recent reviews have been written focusing on different aspects of bioinformatics analyses of IDPs such as the discovery of degenerate motifs in IDPs [62], predicting function in IDPs [63], and the prediction of IDPs by protein sequence [64]. Indeed, the computational tools used to analyze IDPs and IDPRs are as vast as the experimental tools.

#### 8.2.1. Sequence Characteristics

Because of the lack of a stable three-dimensional structure, the computational study of IDPs and IDPRs is predominantly dependent on primary sequence information. Anfinsen’s dogma suggests that the three-dimensional structure of a protein is encoded into the primary sequence [65], however, the accurate prediction of the folded structure from primary sequence remains elusive. Tools to predict disorder from primary sequences have been much more successful, however. This is intuitive from the perspective of entropy. 

**Figure 6:**
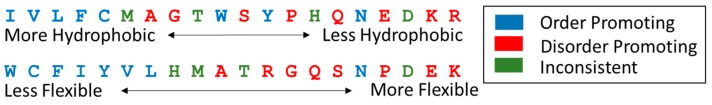
Amino acid scales and disorder and order promoting residues. (**Top**) Ranking of the 20 amino acids by the Kyte-Doolittle hydrophobicity scale from most to least hydrophobic; (**Bottom**) Ranking of the amino acids from most to least flexible by Vihinen’s flexibility scale.

A three-dimensional structure has only one form that it can take, whereas the conformational fluctuations that can define a disordered protein are nearly infinite in their possibilities within steric limitations; therefore, predictors of disorder require less information than predictors of structure. IDPs have distinct sequence characteristics that facilitate the identification of disorder from sequence. IDPs are enriched in specific disorder promoting residues, such as alanine, glycine, serine, proline, glutamine, glutamic acid, lysine and arginine, and they are depleted in the order promoting residues isoleucine, valine, leucine, phenylalanine, cysteine, tryptophan, tyrosine, and asparagine [66,67]. These residues are roughly correlated with flexibility [68] and hydrophobicity scales [69] (Figure 6).

Low complexity regions are often disordered, and disordered regions are often enriched in low complexity motifs. However, neither disorder nor low complexity necessarily implies the other [70]. Disordered regions tend to have lower sequence conservation in families, however, there are also well conserved disordered domains [71]. Furthermore, in poorly conserved disordered regions, the chemical composition is often preserved [72,73].

#### 8.2.2. Disorder Prediction

The distinct sequence features that are present in IDPs and IDPRs allow the construction of sequence based rules that can facilitate high performance disorder prediction. Over 70 predictors of disorder have been created since 1997 [73,74]. A favorable balance between true positives (TP) / true negatives (TN) and false positives (FP)/false negatives (FN) is the objective, and this is typically expressed by the Matthews Correlation Coefficient (MCC).

$$MCC=\frac{TP*TN-FP*FN}{\sqrt{\left(TP+FP\right)\left(TN+FP\right)\left(TP+FN\right)\left(TN+FN\right)}}$$

The CASP competition judges disorder predictors based on as yet unpublished disordered regions, which are usually obtained from missing regions in newly published X-ray crystal structures [75]. The highest ranking predictors in the CASP experiment have an MCC of approximately 0.5 and these results are usually achieved by slower predictors that use multiple sequence alignments along with sequence based features such as amino acid composition and the physicochemical properties of the amino acids. These slow but high performing predictors, such as PrDOS [76], SPINE-D [77], and DISOPRED3 [78], are best for small datasets and single protein prediction. Large datasets, however, require the use of faster predictors that can be run on a local computer, such as Espritz [79], and IUPred [80,81].

Disorder predictors must be trained and tested on datasets of experimentally indicated disordered residues. These datasets commonly come from Disprot [82], X-ray crystal structures in the PDB [83], or NMR data. Most disorder predictors will give a per-residue disorder score between 0.0 and 1.0, and the generally agreed upon threshold for disorder is greater than or equal to a score of 0.5. Some disorder predictors, such as SLIDER [84] or RAPID [85], will provide fast prediction for complete proteomes, by calculating a single score across the entire protein. Other predictors may provide a score that is calibrated differently, such as DynaMine which produces scores in the form of backbone N-H S^2^ order parameter values [55,86]. When using DynaMine, a score below 0.7 is considered flexible.

Meta-predictors, which combine the outputs from multiple single predictors are also common, such as PONDR-FIT [87] and MetaDisorder [88]. A consensus of multiple predictors can provide improved results [89], as it will theoretically reduce the bias inherent in single predictors that were trained on limited datasets. However, despite a modest improvement through consensus methods, disorder prediction based on currently available datasets has likely hit a bottleneck in terms of the maximum possible MCC scores. 

This limitation arises in part due to the imperfections in the testing and training sets for the development of disorder prediction. Experimental indications of disorder are gathered over a wide range of experimental techniques, including low resolution techniques such as circular dichroism which may not provide accurate estimates of the exact disordered residues, X-ray crystallography, which only provides an indication of disorder, but may be caused by other factors, and NMR, which requires significant, and therefore variable interpretation in order to assign disorder. A second issue, which further compounds this, is the presence of conditionally and partially disordered regions, which may be assigned as ordered or disordered, depending on the experiment. Finally, it is likely that there are different flavors of disorder [90] with different sequence based markers, yet a clear classification scheme has not yet been created. 

Disorder prediction should be employed in analysis with an appropriate awareness of the inherent level of error. However, despite these considerations, disorder prediction still provides a way to separate unique sequence regions that indicate a propensity towards disorder, and provides a useful level of biological accuracy, especially at the proteome level. While individual residues may not be assigned correctly in all cases, disorder prediction still provides an illuminating look into the propensity of the protein to be solvent exposed, to undergo dynamic transitions, or to be destabilized by environmental factors. 

#### 8.2.3. Classification of Functions

There appears to be a relationship between biophysical function, cellular function, and sequence characteristics, however, the identification and development of these relationships is still in its early stages [91]. Therefore, a major task in bioinformatics is to attach biological sequence information to physical behavior, biological functions, and cellular response. Functional classification based on sequence requires two sets of information. The first is sequence based features. These may be disorder prediction scores, calculations based on the physicochemical features, or amino acid motifs. The second set of information is functional annotation. Gene Ontology (GO) term assignments are one of the primary sources of annotations related to cellular components, biological processes, and molecular functions [92]. GO terms can be assigned based on experimental evidence, or can be inferred based on homology. Additionally, Enzyme Commission (EC) numbers provide a useful annotation protocol when classifying enzymes [93]. EC numbers are assigned based on the chemical reaction that is catalyzed by the enzyme. Similar to GO terms, EC designations can be made based on direct experimental evidence or can be inferred through sequence homology. 

#### 8.2.4. Proteome Level Studies

The early assumption in protein science was that protein intrinsic disorder represented an unusual and isolated phenomenon. In fact, it can be argued that this assumption is still held by many researchers today [18]. Therefore, the application of disorder prediction to whole proteomes has been critical to establishing the ubiquity and relevance of protein intrinsic disorder while the tools for large scale experimental identification are still nascent. Despite differences in the disorder predictors used, the proteomes they have been applied to, and the different measures applied, the consensus is that eukaryotes tend to have more predicted disorder than prokaryotes or archaea [49], and within eukaryotic proteomes especially, intrinsic disorder is exceptionally common [94]. For instance, Ward et al. found that 2.0% of archaean, 4.2% of eubacterial and 33.0% of eukaryotic proteins had disordered regions greater than 30 residues in length [95]. Estimates of the average fraction of disorder for eukaryotic proteomes tends to be between 20% and 30%, while prokaryotes tend to be closer to 5%–10%, however, there is significant variation and overlap in disorder prediction between the taxa [49,94].

## 9. Protein Intrinsic Disorder and Disease

In 2008, Uversky et al. introduced the disorder in disorders (D^2^) concept, and showed that proteins with IDPRs greater than 30 residues in length are overrepresented in proteins involved with signaling, cancer, neurodegenerative diseases, cardiovascular diseases, and diabetes [96]. While this relationship may suggest innate pathogenicity in IDPs and IDPRs, studies suggest instead that IDPs and proteins with IDPRs perform tightly regulated [97] and necessary functions, many of which depend on the lack of a three-dimensional structure [98]. However, as is the case in structured proteins, genetic, environmental, or systemic perturbations can make IDPRs and IDPRs susceptible to misfolding and misregulation. 

Some of the most well-known examples of disease related IDPs are implicated in neurodegeneration, such as tau, α-synuclein, beta amyloid, and prion protein [99]. Flexibility in these proteins can facilitate perturbations into misfolded, aggregated states, and therefore the disease state is directly related to a structural transition facilitated by the disordered properties of the protein. However, there are also a myriad of potential roles that IDPs and IDPRs can play in disease processes. The pathogenic behavior of an IDP or protein with an IDPR may be triggered by genetic factors such as pathogenic mutations, alternative transcription, or aberrant splicing, or non-genetic factors such as altered protein expression levels, PTMs, or aberrant cleavage. These cellular changes may result in misfolding, loss of normal function, gain of toxic function, protein aggregation, misidentification, misregulation, or missignaling (reviewed in [96] and [100]). 

While uncommon, there are some interesting examples of disease associated enzyme IDPs in the literature. For instance, virulence factors with catalytic domains may utilize long IDPRs to translocate or avoid host defense systems. The adenylate cyclase toxin in *Bordetella pertussis* provides an interesting example of this phenomenon. The adenylate cyclase toxin contains a Repeat in ToXin (RTX) motif, which is intrinsically disordered in the absence of calcium inside the bacterial cell. 

**Figure 7:**
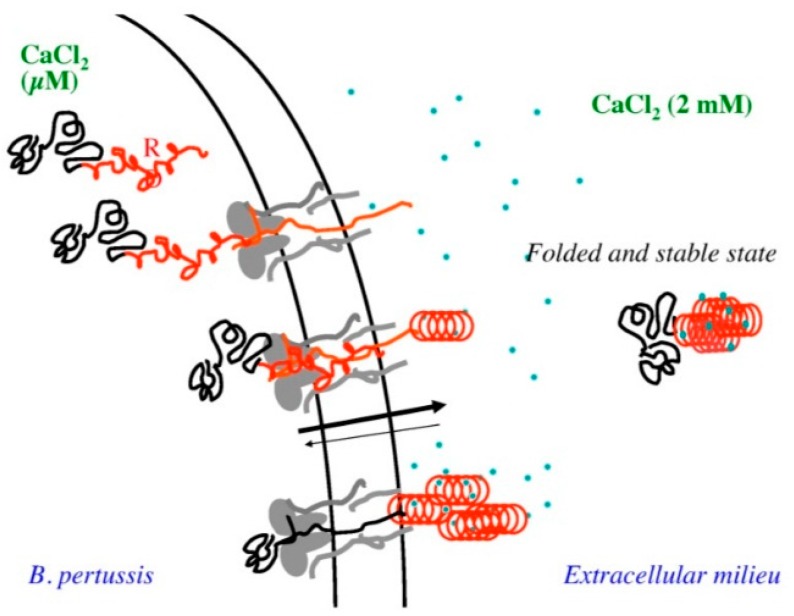
A schematic representation of the secretion of adenylate cyclase toxin through the type 1 secretion system. Reprinted under the creative commons license from ref. [101].

This allows the enzyme to translocate the catalytic domain across the narrow type 1 secretion channel, and then transition to a globular structure when exposed to the calcium gradient on the bacterial cell wall [101] (Figure 7).

The human acetylcholinesterase variant, AChE-R, provides an interesting counter point to IDPR associated pathology. The AChE-R variant has an intrinsically disordered C-terminus, and the presence of this disordered region appears to provide neuroprotective effects in Alzheimer’s disease as compared to the AChE-S variant, which has a helical C-terminus, and appears to accelerate the formation of amyloid fibrils [102]. It is likely that many examples of enzymes with IDPRs that are involved in disease processes in both pathogenic and protective capacities will emerge with the increased acceptance of the functional roles that IDPRs in enzymes may play.

## 10. Protein Intrinsic Disorder and Drug Design and Discovery

The enrichment of long IDPRs in proteins associated with disease processes presents a rich source of potential drug targets, however, there are several challenges in drug design and discovery when IDPRs are targets. Secure binding to a small molecule requires stabilization in the binding site, and this may not be possible in all IDPRs. However, functional IDPRs will often take on a transient structure due to binding or environmental factors which suggests that inducible structure may be possible in many cases. 

In proteins that are mostly disordered in their native state, the disorder can be understood as a population of many interconverting conformations, with the potential to stabilize a single non-pathogenic conformation. The investigation of natural compounds with known effects on IDPs can provide a powerful route to discovery. For instance, the consumption of coffee appears to provide some protection against the development of Parkinson’s disease.

**Figure 8:**
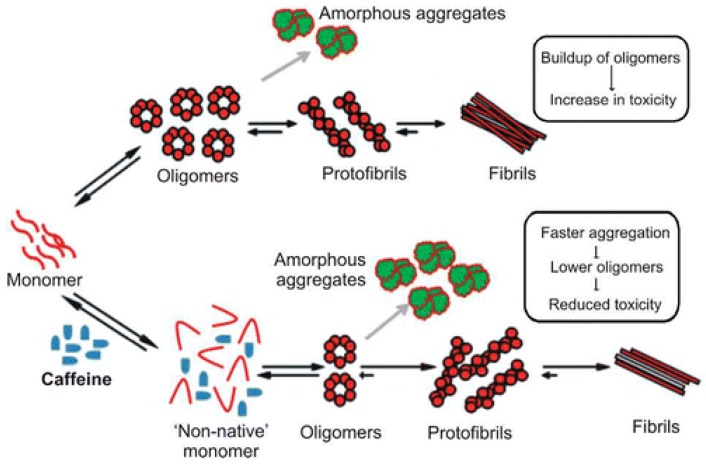
A schematic representation of the effects of caffeine on the aggregation properties of α-synuclein. Reprinted with permission from ref. [103]. Copyright (2015) American Chemical Society.

A study on the effects of caffeine on α-synuclein aggregation showed that caffeine modifies the conformation of the monomer form, thus accelerating the aggregation of a less toxic species [103] (Figure 8). 

Drug design and discovery in IDPRs may be more akin to navigating a handshake than placing a lock in a key, however, many of the principles of design and discovery are still the same. However, when stabilizing binding partners are not known, a clear challenge is the absence of a priori knowledge of the three-dimensional structure for in silico screening or rational drug design of favorable compounds. However, blind exploratory assays are standard in drug discovery, and the presence of an IDPR should not prohibit these kinds of screens. Instead, the biggest challenge in drug discovery for IDPRs may be the standard practice of the exclusion of IDPRs before drug screens.

### The Story of PTP1B

**Figure 9:**
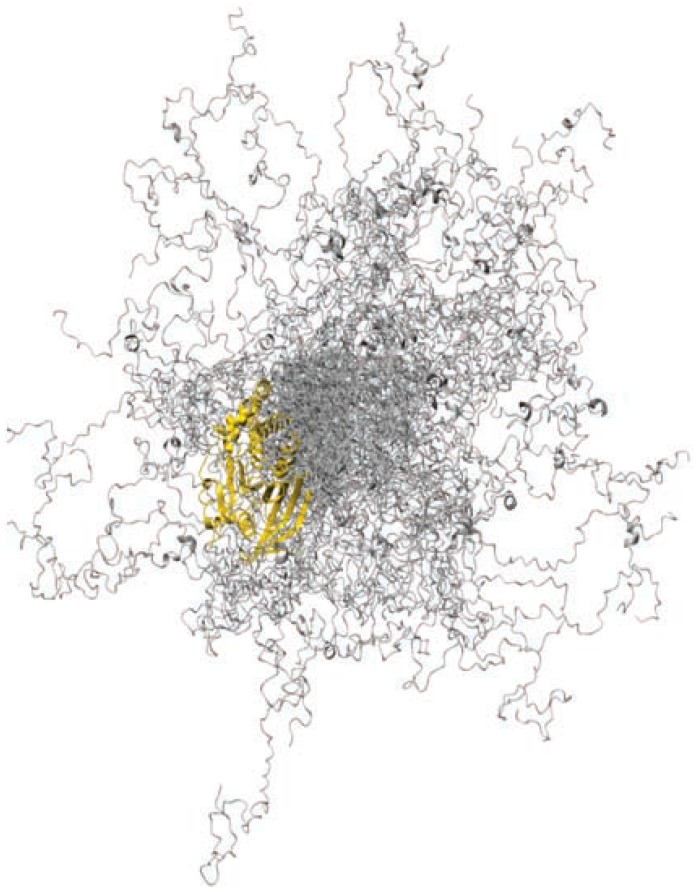
A representative ensemble of 100 conformers for PTP1B. Reprinted with permission from Macmillan Publishers Ltd.: Nature Chemical Biology, part of Springer Nature. [104].

The phosphatase PTP1B (Figure 9) provides an illustrative example of delayed drug discovery for an IDPR due to the consistent truncation of the region before drug screens. 

The catalytic region of PTP1B, encompassing residues 1–321 was purified in 1988 from the human placenta [105], and in 1990 the full length form of 435 residues was uncovered through cDNA cloning [106]. Despite knowledge of the full length form, and an early demonstration of its role in the regulation of PTP1B [107], studies on PTP1B between 1990 and 2014 focused almost exclusively on the originally purified form encompassing residues 1–321, therefore ignoring the disordered C-terminal region. 

PTP1B became an attractive therapeutic target due to its involvement in multiple signaling pathways, including those implicated in obesity and diabetes [108]. However, the development of a small molecule inhibitor for the catalytic domain of PTP1B was frustrated by the highly charged nature of the catalytic site [109], and the practice of testing inhibitors against the truncated form. It was not until 2014 that MSI-1436, a known inhibitor of PTP1B in vivo [110], was screened against the full length form, and was found to be an effective inhibitor [104]. 

The C-terminal region of PTP1B is intrinsically disordered, moving within a wide range of three-dimensional space (Figure 9). However, as is often the case in IDPRs, there is residual secondary structure in the form of two small α-helical regions. The most peripheral of these αhelical regions provides one anchor point for MSI-1436, while the second anchor point is found close to the catalytic domain. Upon binding to these two regions, PTP1B becomes more compact, and V_max_ is decreased.

While MSI-1436 provides a small amount of inhibition of the truncated form of the enzyme, the primary biding site is between residues 367 and 394, and therefore this region is required to observe the full strength of the inhibition. To our knowledge, this was the first drug screen against the full length form; therefore, it is possible that MSI-1436 or other effective inhibitors had been tried and discarded previously.

The story of PTP1B demonstrates that it is sometimes assumptions about the lack of functional relevance of IDPRs in enzymes that creates one of the largest obstacles to understanding and utilizing these regions in disease intervention.

## 11. The Field of Protein Intrinsic Disorder

An un-structural biologist specializes in the tools and techniques used to study IDPs. Furthermore, a specialist in the field of IDPs must be aware of individual proteins identified as IDPs and the body of experimental, proteomic and bioinformatics literature validating the existence of disorder in these proteins. Due to the broad scope of the material covered by the field of protein intrinsic disorder, and the nascence and relative obscurity of the field, there are a number of researchers who focus almost exclusively on the study of IDPs from various perspectives. 

**Figure 10:**
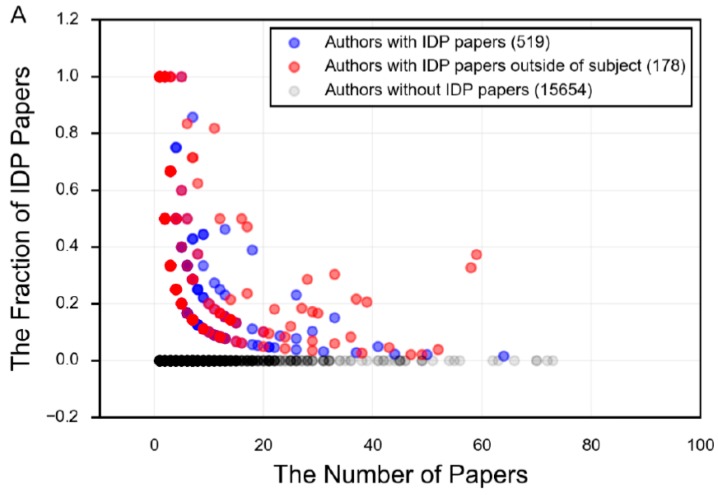

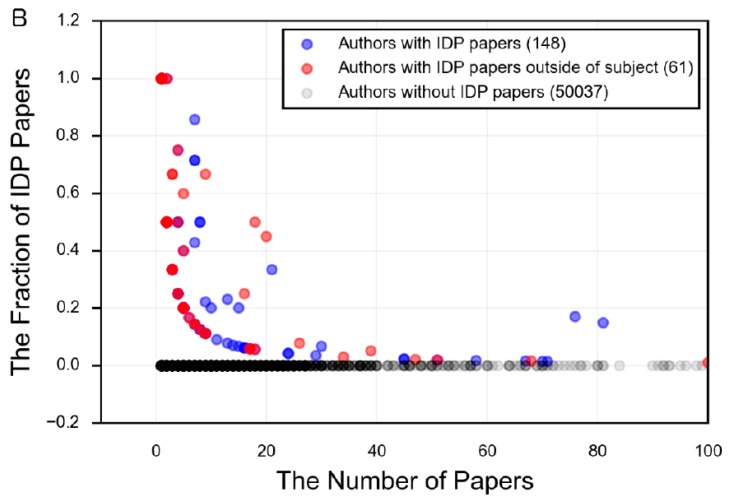
The number of papers per author for the search term in PubMed, plotted against the fraction of those papers that use IDP terminology. Each point represents an author on one or more papers associated with the given search term. The darker the dot, the larger the concentration of authors at that point. Blue dots are authors who have an IDP paper in the field in question (α-synuclein or tau, in this case), while the red dots are authors who have an IDP paper in the field in question and also have an IDP paper in a different field. The fraction of IDP papers is the number of papers by that author that use IDP terminology divided by all papers for that author and search term. The following search terms were used: (**A**) “alpha synuclein”; (**B**) “tau AND (protein OR Alzheimer’s OR tauopathies OR neuronal)”.

The field of protein intrinsic disorder represents a powerful example of the productive relationship between experimental and bioinformatics techniques (Figure 5). For example, an experimentalist who notices unusual structural behavior in their protein may employ the use of disorder prediction to assess the propensity of their protein towards disorder. Using this information as a guide, they can target their research towards those regions predicted to be intrinsically disordered, and apply the appropriate experimental techniques. This experimental data yields information, which can then be used to revise and improve the prediction of disorder in other proteins, to extrapolate evolutionary information for the protein family, or to predict function or biophysical mechanisms. However, the number of researchers who specialize in the study of *specific* proteins that are intrinsically disordered, and who also embrace the language of disorder to describe these properties, is remarkably small (Figure 10), demonstrating that the tools and language of protein intrinsic disorder has not propagated far beyond those who specialize in studying IDPs.

## 12. Conclusions: Intrinsic Disorder Where You Least Expect It

As this new paradigm of un-structural biology becomes more accepted, it becomes clear that the disciplines of structural and un-structural biology can work in tandem to explain the dynamics of a protein over time. One should keep in mind though that from the viewpoint of complete lack of structure and complete sequence randomness, the structure-function relations in IDPs and IDPRs, where the structure is defined as a “low-resolution” ensemble of conformations, where the IDP/IDPR sequences are not random, and where IDPs/IDPRs have specific functions, the phrase “un-structural” biology does sound like a stretch. However, from the viewpoint of traditional “structural biology” that still serves as an operational term for many researchers working in the field of protein science, the aforementioned “low-resolution” ensemble of conformations cannot be considered as “unique structure” needed for a protein to have “unique function”. However, structural and functional characterization of ordered and disordered proteins does require very different methodological approaches, thus justifying and supporting the existence of two very different schools of thoughts. On the other hand, opposing “structural” and “un-structural” biology is counter-productive. In our view, these two concepts should not be opposed, but united, since they clearly complement each other. Furthermore, the line between order and disorder is a practical line, and the language and tools of protein intrinsic disorder become necessary when a protein or protein region can no longer be described in three dimensions. The acceptance of the presence and functional relevance of protein intrinsic disorder, however, remains relatively low, especially in protein populations that are expected to be structured. Therefore, this review highlights several challenges:

- There is a focus in the IDP literature on mostly or fully disordered proteins, and an incomplete understanding of the diverse mechanisms and functions employed by proteins that have regions of both order and disorder.
- The development and validation of disorder prediction depends on experimental datasets of missing regions from the PDB, however, it is not always clear what the cause and nature of the missing region is.
- There is an increasing number of enzymes with experimentally measured IDPRs in the literature, but no proteome level studies up to this point.
- There is limited acceptance of the language of protein intrinsic disorder outside of those who specialize in studying IDPs.
- Drug design and discovery frequently focuses on the truncated form of the protein, potentially resulting in missed opportunities.

Furthermore, it seems that future studies should be focused on addressing the question of how likely IDPRs are to occur in canonically structured proteins, and whether and how these regions are functionally significant.
